# Ultrasound-guided fascial plane blocks in chronic pain: a narrative review

**DOI:** 10.1186/s44158-024-00205-y

**Published:** 2024-10-16

**Authors:** Francesco Marrone, Carmine Pullano, Alessandro De Cassai, Pierfrancesco Fusco

**Affiliations:** 1 Anesthesia and Intensive Care, Santo Spirito Hospital, Rome, Italy; 2 Anesthesia, Villa Pia Clinic, Rome, Italy; 3 Sant’Antonio Anesthesia and Intensive Care Unit, University Hospital of Padua, Padua, Italy; 4 Anesthesia and Intensive Care, SS Filippo E Nicola Hospital, Avezzano, L’Aquila Italy

**Keywords:** Fascia, Nerve blocks, Erector spinae plane, Pain management

## Abstract

**Background:**

Recent studies have unveiled the intricate and distinctive nature of fascia, no longer regarding it solely as a muscle container. Recent research highlights its complex innervation and structure, signifying its significance in chronic pain pathways.

**Methods:**

We conducted a systematic literature search (updated on February 2024) to evaluate the role of fascial plane blocks in chronic pain treatment. All article types (randomized clinical trials, prospective and retrospective observational studies along with case reports and case series) were deemed eligible for inclusion if they referenced “fascial plane blocks” for the control of chronic pain conditions (persistent post-surgical, neuropathic, musculoskeletal-myofascial and cancer-related) and were published between 2010 and February 2024.

**Results:**

The search revealed an increasing evidence in the literature for the implementation of fascial blocks in chronic pain management, although still heavily limited to case reports or series.

**Conclusion:**

With the integration of ultrasound technology and a deeper understanding of their mechanisms of action, the fascial plane blocks continue to broaden their application also in chronic pain management, as a part of a multimodal strategy or as an alternative to conventional drugs or opioids.

## Introduction

Chronic pain is a continuing adversary that often defies traditional remedies and causes profound disruptions in daily life [1]. Chronic pain may be defined as persistent pain for more than 3 months. The proposed classification system differentiates between different kinds of chronic painful disorders. It comprises many conditions associated with persistent or recurrent pain, according to the classification of chronic pain in the ICD-11 [2, 3].

People troubled with chronic pain are continuously looking for alleviation, turning to a myriad of treatment plans and interventions in hopes of locating respite. Opioids are indispensable for the treatment of severe pain. However, the (over)prescribing of high doses of opioids, mostly for chronic pain, led to unacceptable rates of complications.

In particular, nerve blocks and new fascial plane blocks (FPBs) have emerged as promising options for the management of chronic aches, resulting in effective options for reducing drug and opioid consumption.

**Fig. 1:**
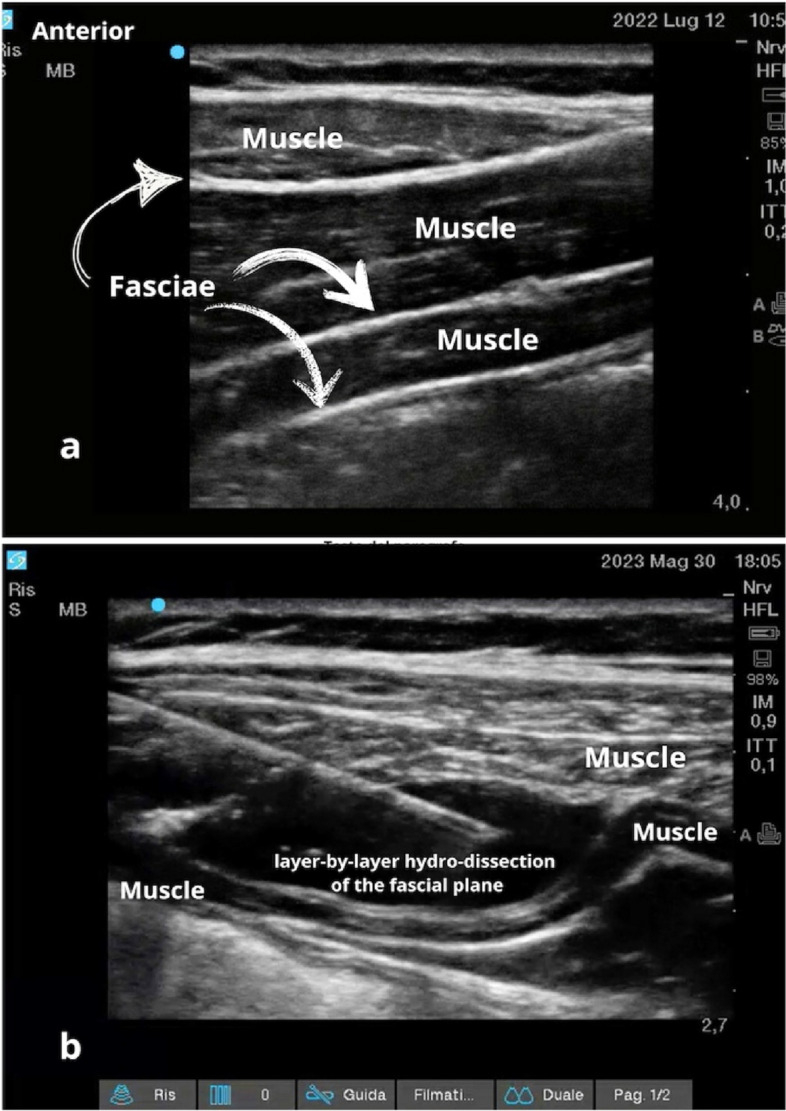
Sonographic images of a fascial plane. **a** Ultrasound scan of the anterior abdominal wall. From top to bottom, the three muscle planes (external oblique, internal oblique, and transversus abdominis muscles) and their respective epimysial fasciae are shown. Note the hyperechogenicity and gliding of the fascial structures due to the presence of loose connective tissue and fat between the fasciae of the three muscles. **b** Ultrasound representation of a dynamic TAP block: the needle, through a hydro-dissection mechanism, opens the layers of the epimysial fascia between the internal oblique and transversus muscles

Fascial plane blocks involve the injection of local anesthetic into a unique space (plane) between two layers of fascia surrounding tissues, muscles, and nerve organizations implicated in the origin of pain [4]. This interventional procedure is usually performed under ultrasound guidance by visualizing the fasciae as thin hyperechoic lines surrounding the muscles and nerves with a small amount of hyperechoic fat tissue between the fascial layers (gliding zones) [5] (Fig. 1).

Different techniques can be used to inject a fascial plane, e.g., in-plane technique vs. out-of-plane technique. The in-plane needling has the significant advantage of allowing the needle to be seen along its entire length, including its tip. This way, the direction of the puncture can be carefully monitored, while also facilitating the opening maneuver of the fascial plane with the needle itself and the injectate. Ultrasound plays a pivotal role in optimizing the safety of fascial plane blocks, allowing to avoid unintentional iatrogenic injuries to vital anatomical structures such as vessels, nerves, or pleura.

Unlike systemic medicinal drugs, which can cause significant side effects and have limited efficacy, fascial plane blocks offer directly targeted alleviation of the site and the mechanisms underlying pain. A key advantage of fascial blocks lies in their potential to disrupt the transmission of nociceptive signals along neural pathways, successfully dampening the belief of the ache. By selectively blocking sensory nerves or modulating neurogenic pathways, these interventions disrupt the pathological pain cycle function of persistent pain syndromes. Furthermore, the extent of the analgesic effect of fascial plane blocks may be prolonged through the use of adjuvants such as corticosteroids or liposomal formulations, enhancing the sustainability of pain treatment.

The procedural component of administering these blocks fosters a sense of empowerment and self-efficacy in patients, as they actively participate in their own remedy process. Moreover, the localized nature of fascial blocks allows clinicians to tailor interventions to the individualized anatomical and physiological traits of each affected person, optimizing results while minimizing the hazards of systemic complications.

Nevertheless, the potential of fascial plane blocks for chronic pain management is not without limitations and challenges. Variability in anatomical structures and in sonographic recognition, the presence of comorbidities, the use of other strategies, variability in affected persons’ responses, and the potential for procedural complications necessitate a comprehensive assessment of hazard-benefit profiles prior to their implementation. Currently, we do not fully know how these blocks work in all settings, and they are not adequately compared with other regional or nonregional techniques. Additionally, the most efficient timing and frequency of block interventions remain a subject of debate, emphasizing the need for further research to clarify their lengthy-term results and scientific application [6].

The intention of our article is to review the present literature concerning the use of principal fascial plane blocks for chronic pain management. While the coverage of numerous plane blocks cannot be exhaustive, the work facilities on the maximum often accomplished interventions in this field, supported through evidence. Additionally, our undertaking included assessing the modern degree of proof helping each person approach. Last, we want to remind readers that the sphere of fascial blocks is continuously advancing and debating, and we inspire them to live informed by searching for continuous updates.

## Materials and methods

All article types (randomized clinical trials [RCTs], prospective and retrospective observational studies along with case reports and case series) were deemed eligible for inclusion if they referenced “fascial plane blocks” for the control of chronic pain conditions (persistent post-surgical, neuropathic, musculoskeletal-myofascial and cancer-related) and were published between 2010 and February 2024. The inclusion criteria for participants were age 18 years and older; fascial plane blocks (erector spinae plane [ESP], including cervical, thoracic, lumbar and sacral blocks]; pectoral blocks [PECs], including interpectoral, pecto-serratus, and serratus plane blocks]; transversus abdominis plane [TAP]; quadratus lumborum [QL]; pericapsular nerve group [PENG] block; fascia iliaca compartment block [FICB]; adductor canal block [ACB]; chronic pain conditions refractory to standard therapy; and pain assessment using standard scales (including visible analog scales [VAS] or numeric score scales [NRS]) before and after procedures. Studies regarding the impact of preoperative fascial plane block implementation on chronic persistent post-surgical development were included. Various healthcare settings (along with scientific facilities, hospitals, and clinics) were considered for inclusion. The exclusion criteria included the absence of clean descriptions of the clinical context and methodology, the use of cadaveric or animal research, or the use of studies involving pediatric patients. This review focused on chronic pain conditions, previous analgesic treatments administered before fascial plane blocks, blockade techniques, pain intensity, analgesic efficacy of FPBs, length of effect, complications, and assessment of the utility of FPBs in persistent pain management.

The following data were extracted: author details, publication year, country, citation, participant characteristics (age, sex, weight, comorbidities), type of pain (persistent post-surgical, neuropathic, musculoskeletal-myofascial and cancer-related), type of block, local anesthetic information (concentration and dose), administration mode (single or continuous), duration, complications, and side effects. A comprehensive evaluation was carried out to distill the primary information from the cases and the authors' conclusions. A literature search was performed through PubMed, CENTRAL, and EMBASE using the keywords “fascial plane blocks”, “erector spinae plane”, “pectoral blocks”, “serratus plane blocks”, “transversus abdominis plane”, “quadratus lumborum”, “PENG” block, “fascia iliaca compartment block”, and “adductor canal block”, with topic headers and free-text terms separated through the Boolean operators AND and OR. Additionally, key references from bibliographies of relevant studies published in English between January 2010 and February 2024 were searched.

### Selection criteria

Initially, based on the title and abstract, and then on the full texts, one reviewer independently assessed the retrieved studies in consideration of the inclusion standards. Another reviewer reaffirmed their inclusion, with any discrepancies resolved through discussion. Technical reports, editorials, animal research, and cadaver studies were excluded due to resource constraints or the unavailability of complete textual content. Studies published in languages other than English were excluded. We checked the quality of the data available on fascial plane blocks and assigned a level of evidence as previously defined by the Centre for Evidence-Based Medicine [7] (Table 1).

**Table 1:** Level of evidence as previously defined by the Centre for Evidence-Based Medicine

1a	Systematic review (with homogeneity) of RCTs
**1b**	Individual RCT (with narrow CIs)
**1c**	All-or-none study
**2a**	Systematic review (with homogeneity) of cohort studies
**2b**	Individual cohort study, including low-quality RCTs (e.g., < 80% follow-up)
**2c**	‘Outcomes’ research; ecological studies
**3a**	Systematic review (with homogeneity) of case–control studies
**3b**	Individual case–control study
**4**	Case series (and poor quality cohort and case-controlled study)
**5**	Expert opinion without explicit critical appraisal or based on physiology, bench research, or ‘first principles’

The review was also conducted with Covidence systematic review software [8]. The authors’ details, publication year, participant characteristics (age, sex, comorbidities), type of pain (persistent post-surgical, neuropathic, musculoskeletal-myofascial and cancer related), type of blocks, local anesthetic details (concentration, dose), administration mode (single or continuous), duration, complications, and side effects are summarized in a specific database to point out some relevant data and highlights of the blocks prior to the discussion.

## Results

**Fig. 2:**
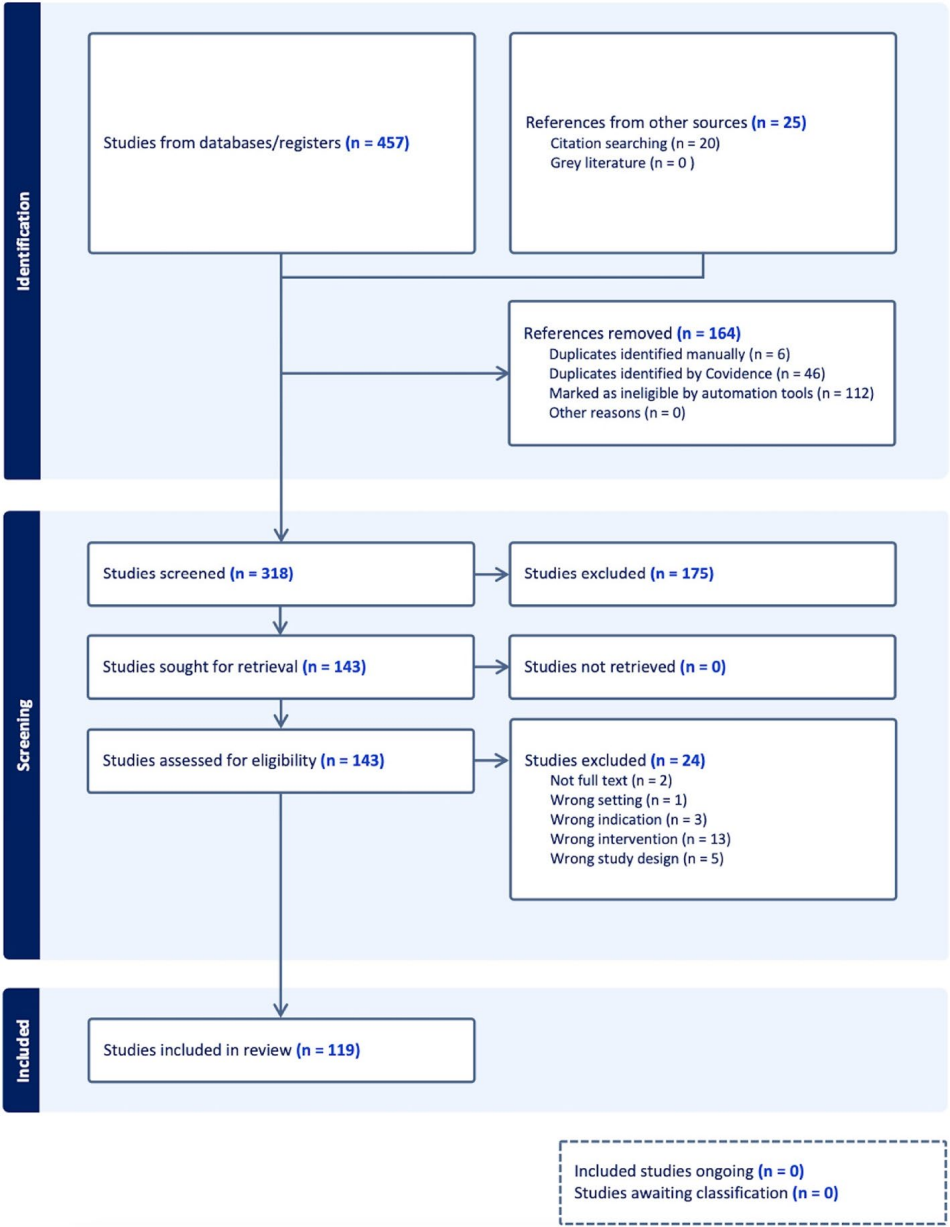
PRISMA flow diagram. *PRISMA*, Preferred Reporting Items for Systematic Reviews and Meta-analyses

A total of 457 articles from databases met the requirements of the preliminary search. Twenty-five studies were added from references from other sources. Among these, 164 studies were removed (duplicates identified manually 6, duplicates identified by Covidence 46, marked as ineligible by automation tools, 112). Then, 318 studies were screened, and 175 were excluded. After reviewing the whole texts of the publications, 143 studies were retrieved and assessed for eligibility. Of these, 24 were excluded (not full-text 2, wrong setting 1, wrong indication 3, wrong intervention 13, and wrong study design 5). Ultimately, 119 papers met the criteria for inclusion in the review (Fig. 2 PRISMA diagram [9]).

The study characteristics (including the type of study, type of pain, and fascial block) are shown in Table 2.

**Table 2:** Type of studies

*Review*	**9**
* Randomized controlled trial*	4 T-ESP, 5 PECs, 1 FIB, 1 PENG, 4 QL, 3 TAP, 1 ACB
* Observational (p, r)*	7 T-ESP, 2 L-ESP, 10 PECs, 1 PENG, 5 QL, 6 TAP, 4 ACB
* Case series-report*	6 L-ESP, 2 S-ESP, 29 T-ESP, 6 PECs, 1 FIB, 2 PENG, 3 QL, 7 TAP
**Type of pain**	
* Post-surgical*	51
* Myofascial-MSK*	25
* Cancer-related*	8
* Neuropathic*	20
* Others*	*1 (vaginismus), 2 (CRPS), 1 (PDPH), 1 (flank pain), 1 (chronic pancreatitis)*
**Type of block**	
* Cervical ESP*	*0*
* Thoracic ESP*	*40 (*+ *6 review)*
* Lumbar ESP*	*8*
* Sacral ESP*	*2*
* PECs*	*21 (*+ *2 review)*
* TAP*	*16*
* QL*	*12 (*+ *1 review)*
* PENG*	*4*
* FIB*	*2*
* ACB*	*5*

Characteristics of the included studies

The level of evidence of the included studies classified according to the type of pain and anatomical district (anterior trunk, posterior trunk, lower extremities) are reported in Table 3 while Table 4 presents only RCTs with details for each block.

**Table 3:**
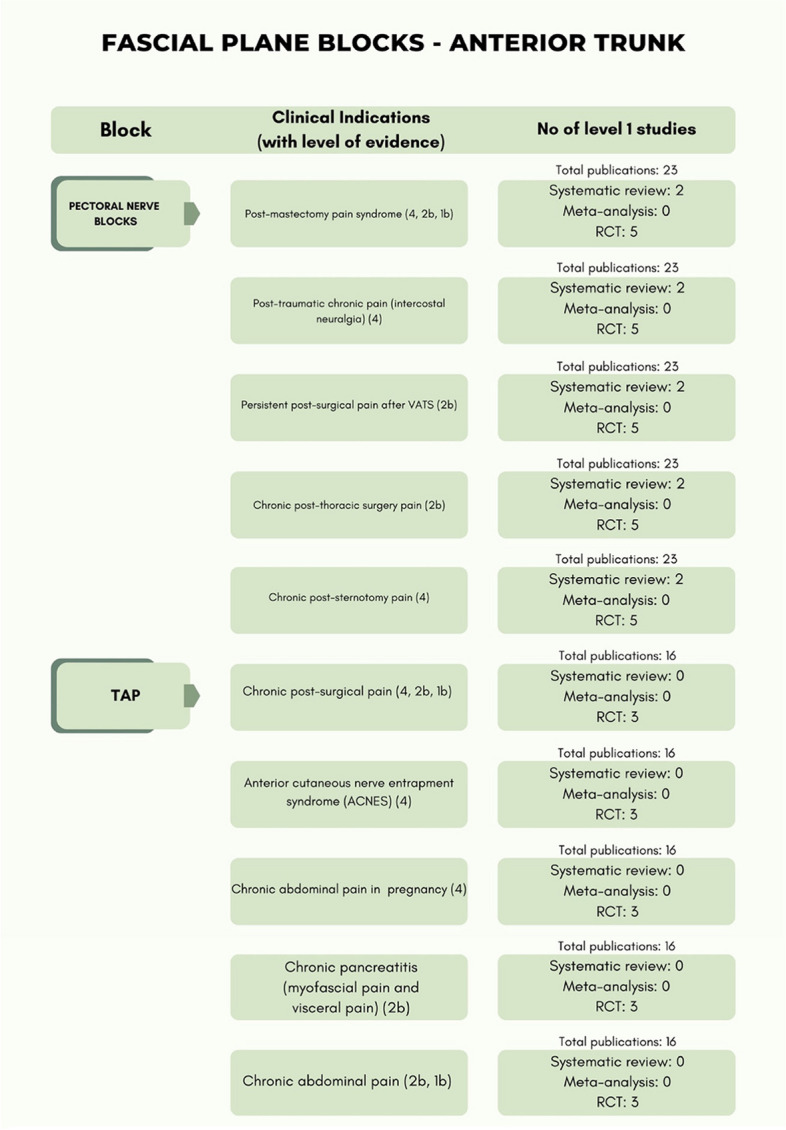

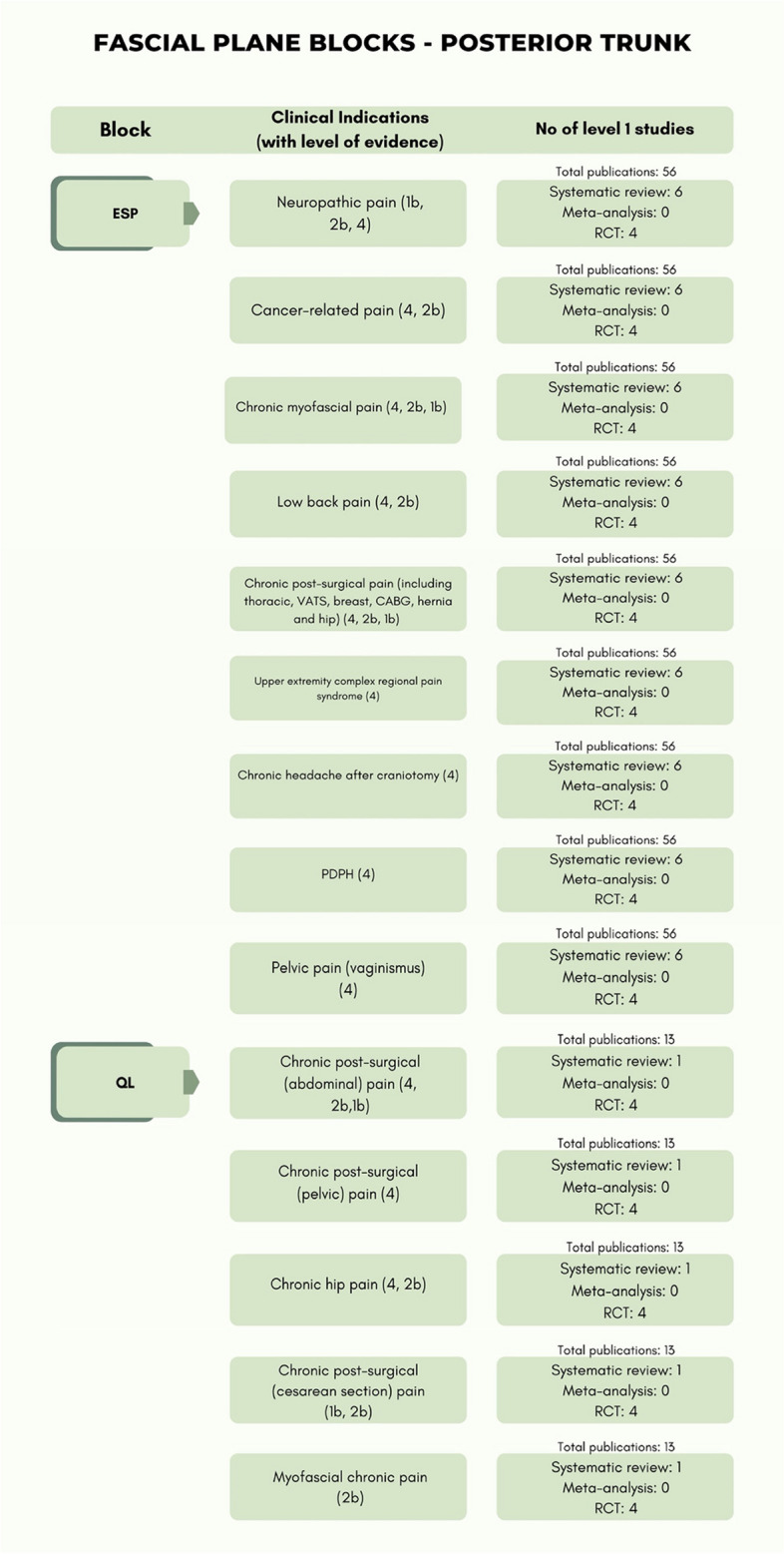

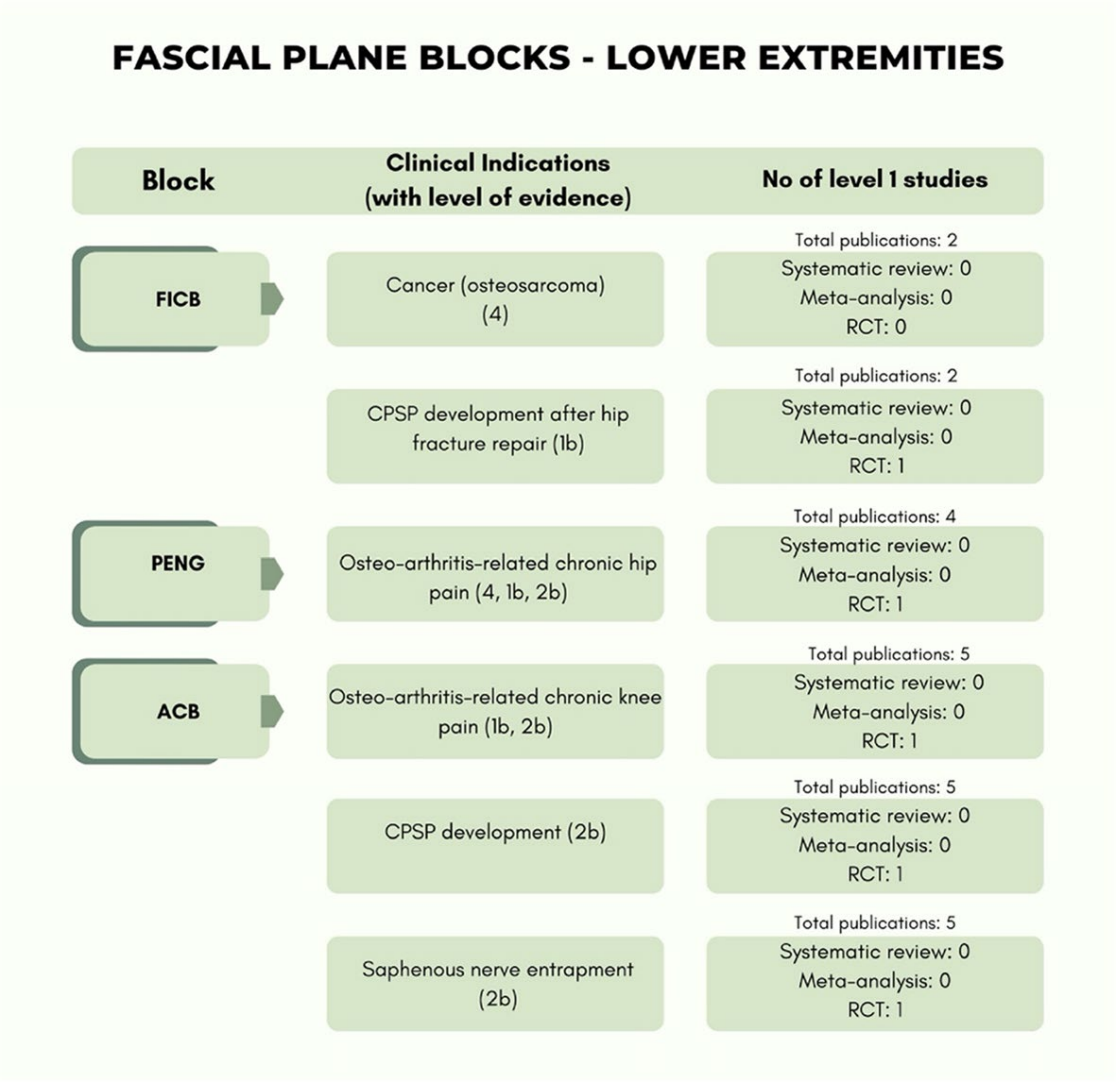
The level of evidence of the included studies classified according to type of pain and anatomical district (anterior trunk, posterior trunk, lower extremities)

**Table 4:**
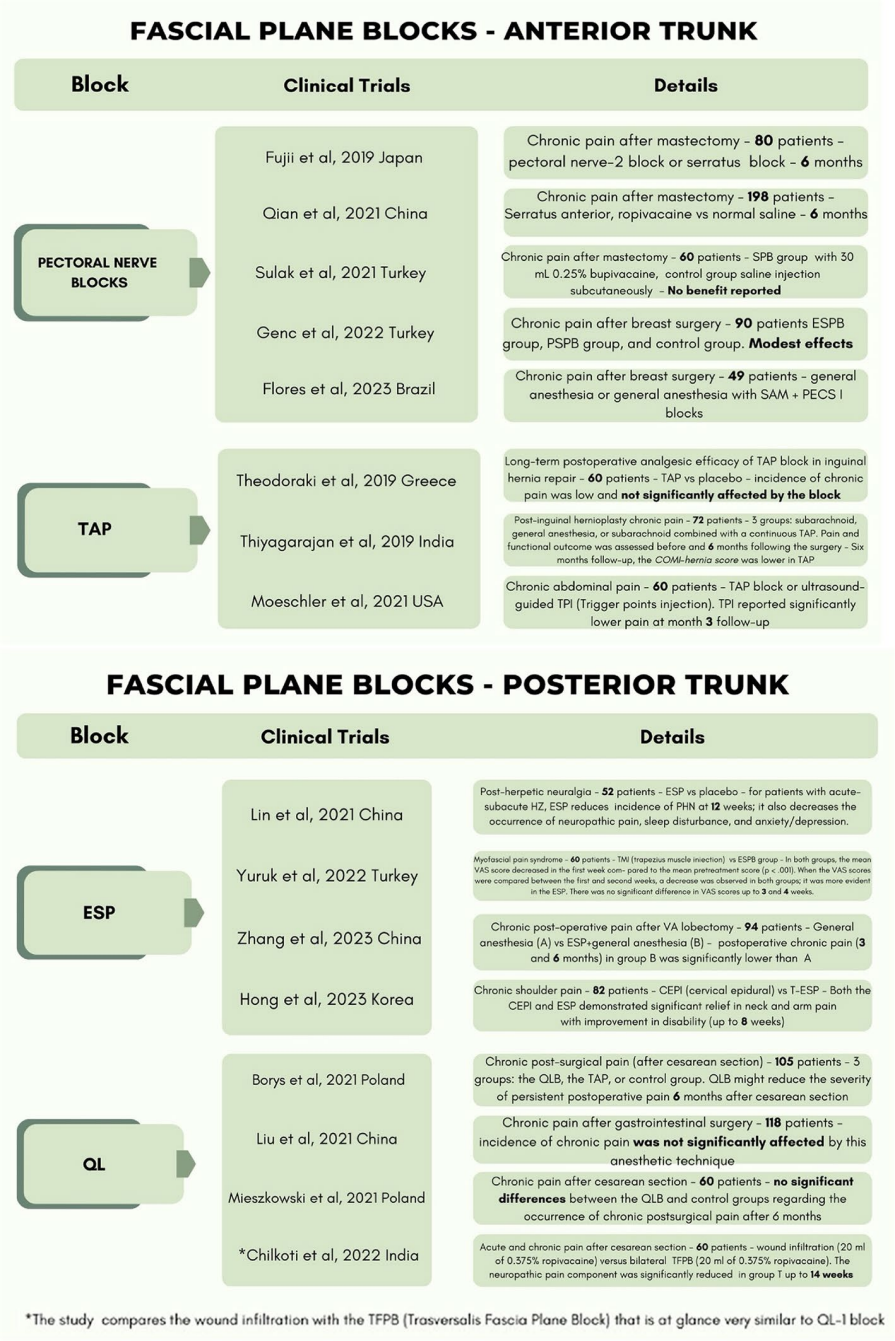

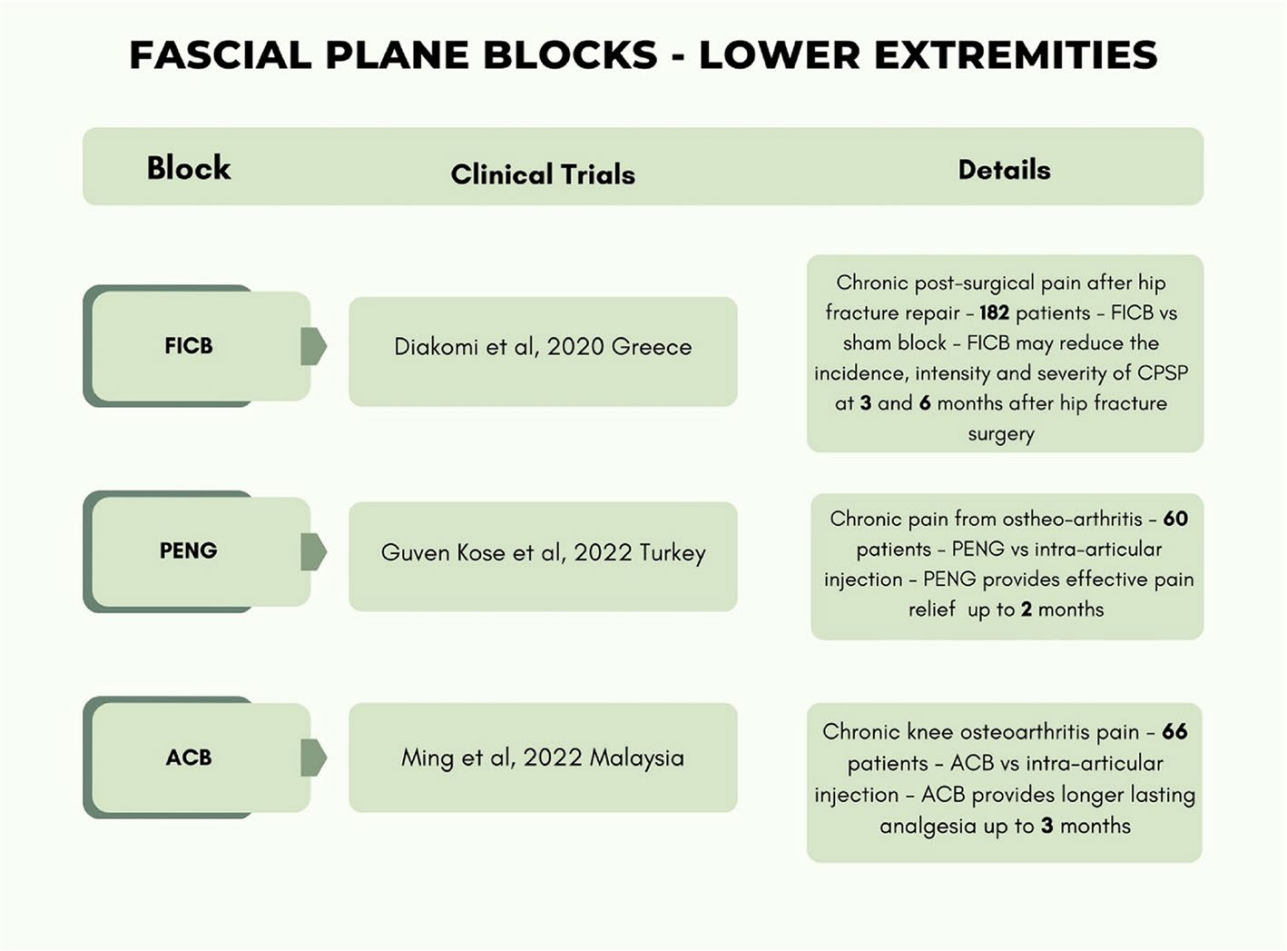
Included RCTs in detail for each block

The different types of (ultrasound-guided) blocks and the anatomical/sonographic landmarks to perform an accurate procedure are summarized in Table 5.

**Table 5:** The different types of (ultrasound-guided) blocks and the anatomical/sonographic landmarks to perform an accurate procedure

**Different types of (ultrasound-guided) blocks**	**Some anatomical/sonographic landmarks**
**Posterior trunk**	
*Erector spinae block (ESP)* *Quadratus lumborum block (QL)*	Probe position: transverse orientation at the midline of the spine to identify the spinous processes. It is then moved laterally until the transverse process is visualized. A linear probe is used for thoracic-level puncture Injection site: in the plane between the erector spinae muscle and transverse process, at T2–L4 levels depending on surgical procedures Dosing: 15–30 mL per block At the lumbar level with the *convex probe* in parasagittal longitudinal approach, injection can be superficial to the transverse processes of the vertebrae along the undersurface of the erector spinal muscle or crossing the intertransverse ligaments with the needle and releasing the drug beneath the transverse processes. Dosing: 20–30 mL per block At the sacral level, with the (linear) probe in longitudinal orientation, the injection can be placed at the median or intermediate sacral crest at S1-S4 levels, under the multifidus muscle. Patient may be placed in prone or lateral position. Dosing: 20–30 mL per block Probe position: patient could be either in lateral or supine position. Convex probe is placed in a transverse, oblique orientation at the L2-L4 level to best identify the QL muscle Injection site: according to ESRA/ASRA consensus^a^, lateral approach with injection in the plane between the aponeuroses of internal oblique and transversus abdominis muscles at the lateral corner of quadratus muscle. Posterior approach with injection on the posterior aspect of quadratus between the posterior QL and erector spinae muscles. Anterior approach when the injection is in the plane between quadratus lumborum and psoas major muscles. Intramuscular injection. Interestingly, the QL block may represent an “intra-muscular injection”: using the cortical bone of iliac crest as a landmark, the needle is advanced inside the muscular belly of the QL muscle Dosing: 20–30 mL per block
**Anterior trunk**	
*Pectoral nerve blocks (PECs)* *Transversus abdominis plane block (TAP)*	Probe position: the patient could be either in the lateral or supine position. A linear probe is placed on the anterior thorax Injection site: according to ESRA/ASRA consensus^a^: Inter-pectoral plane (IPP) block, between the two pectoral muscles (major and minor); Pecto-serratus plane (PSP) block, with the injection between pectoralis minor and serratus anterior muscles; and Serratus anterior plane (SAP) block, deep when the injection occurs in the plane under the serratus muscle at the contact with rib or superficial when injection is placed superficial to serratus anterior muscle Dosing: 15–25 mL per block Probe position: the patient could be either in the lateral or supine position. A linear probe is placed on the anterior abdomen Injection site: according to ESRA/ASRA consensus^a^, injection in the plane between the internal oblique and transversus abdominis muscles. The injection may be placed also at the midaxillary line or at the subcostal level, in the plane between the internal oblique and transversus along the medial costal margins in the upper quadrants of the anterior abdominal wall Dosing: 20 mL per block
**Lower extremities**	
*Fascia iliaca compartment block (FICB)*b *Pericapsular nerve group block (PENG)* *Adductor canal block (ACB)*	Probe position: transversely at the inguinal ligament. Identifying the iliacus muscle, iliopsoas muscle, fascia iliaca, femoral artery, femoral nerve Injection site: the needle is placed in the plane (lateral to medial) deep to the fascia iliaca, above the iliacus muscle. Dosing: 30–40 mL per block Probe position: below the ASIS at 45° to align with the pubic ramus; identifying the AIIS, IPE, Iliopsoas tendon, and femoral artery. Injection site: lateral to medial, needle tip lateral, and below the iliopsoas tendon. According to ESRA/ASRA consensus^c^ Injection is placed in the musculofascial plane between the psoas tendon anteriorly and the pubic ramus posteriorly. Dosing: 20–30 mL per block. For injectate > 15 mL motor weakness has been reported Probe position: transverse, identify sartorius, vastus medialis, adductor longus muscles, femoral artery, saphenous nerve Injection site: in-plane, lateral to medial, tip at anterolateral aspect of femoral artery, distal to the apex of the femoral triangle, and proximal to the adductor hiatus. Dosing: 15–20 mL per block

^a^El-Boghdadly K, Wolmarans M, Stengel AD, Albrecht E, et al. Standardizing nomenclature in regional anesthesia: an ASRA-ESRA Delphi consensus study of abdominal wall, paraspinal, and chest wall blocks. Reg Anesth Pain Med. 2021 Jul;46(7):571–580

^b^Only the infra-inguinal approach is described, following the technique of the included studies

^c^El-Boghdadly K, Albrecht E, Wolmarans M*,* et al.Standardizing nomenclature in regional anesthesia: an ASRA-ESRA Delphi consensus study of upper and lower limb nerve blocks

Reg Anesth Pain Med. 2023 Published Online First: 22 November 2023. 10.1136/rapm-2023-104884.

*ASIS* Anterior superior iliac spine, *AIIS* Anterior inferior, iliac spine, *IPE* Ilio-pubic eminence

Different types of ultrasound blocks with some relevant anatomical and sonographic landmarks to perform an accurate procedure.

**Fig. 3:**
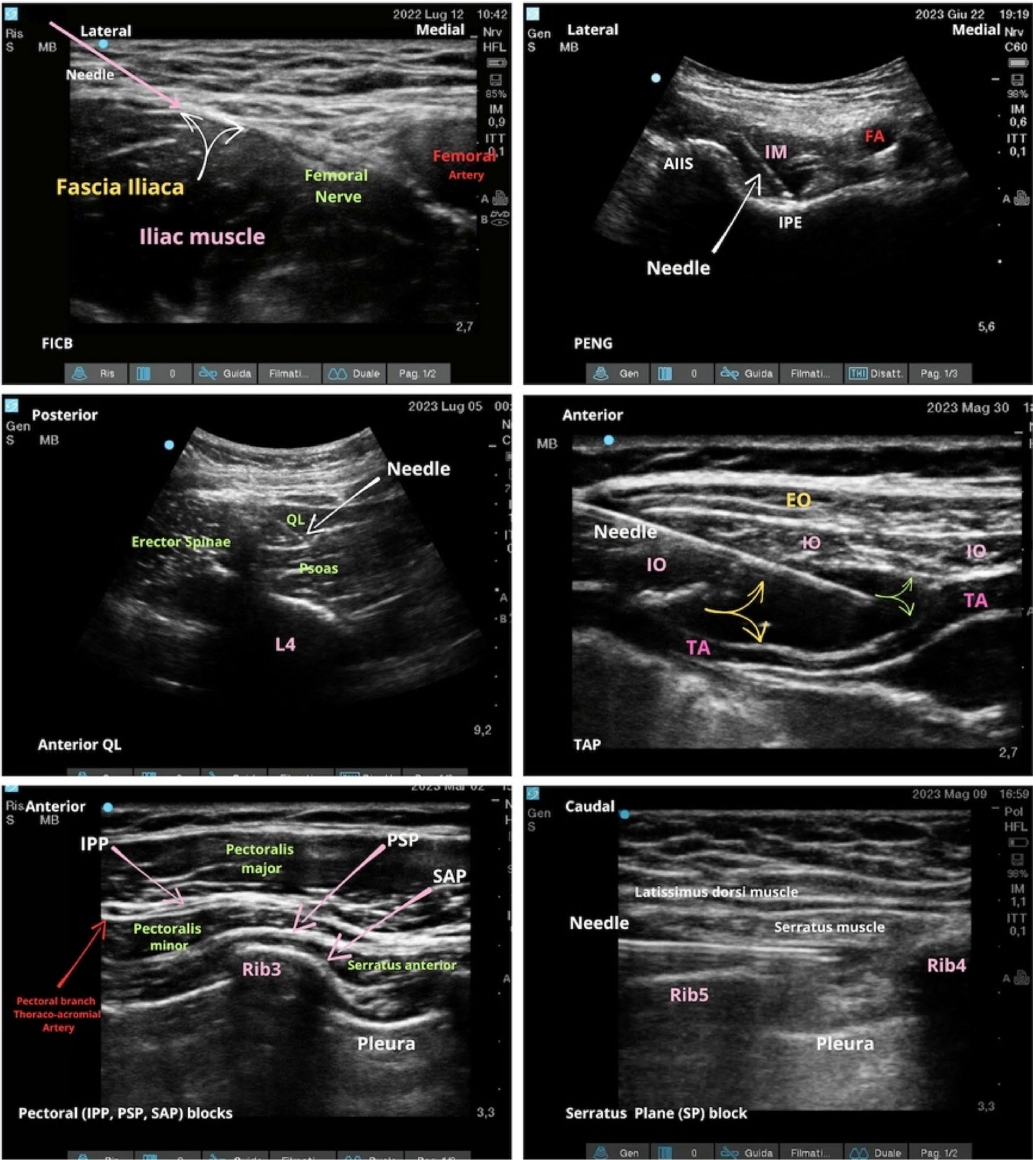
Different types of fascial plane blocks are shown together with relevant sonoanatomy and approaches. FICB, fascia iliaca compartment block; AIIS, anterior inferior iliac spine; IM, iliac muscle; IPE, Ilio-pubic eminence; FA, femoral artery; QL, quadratus lumborum; EO, external oblique muscle; IO, internal oblique muscle; TA, transversus abdominis muscle; TAP, transversus abdominis plane; IPP, inter-pectoral plane; PSP, pecto-serratus plane; SAP, serratus anterior plane

The different types of fascial plane blocks with relevant anatomy and approaches are depicted in Fig. 3.

## Posterior truncal blocks

### Erector spinae plane block

#### Literature review

At the time of this writing, there were more than 50 publications on the use of the erector spinae plane for chronic pain, comprising 6 systemic reviews, 4 randomized controlled trials (RCTs), and numerous case reports. Among the reviews, the most recent one by Ferreira-Silva [10] discusses the usefulness of ultrasound-guided procedures in the treatment of chronic thoracic back pain. This technical review delves into 5 ultrasound-guided thoracic interventions, including ESP block, providing a detailed technical description. De Cassai et al. [11] advocated for the utility of ESP block for chronic pain based on the literature, highlighting its indications for neuropathic pain, myofascial pain, and radiculopathies. They emphasize its low complication rates, the ease of execution, and the versatility of indications. In the face of growing popularity, the authors caution about long-term effects, infection risks, combination with other therapies, and the necessity for a deeper understanding of mechanisms.

Other reviews also concur on the safety and ease of ESP block in chronic pain, a problem that is reaching pandemic proportions. Viderman et al. [12] underscore the need to improve the quality of studies supporting the use of ESP in chronic pain, as they mostly consist of case reports.

With regard to the studies supporting ESP block in chronic pain, it is noteworthy that the first description of ESP block pertained to neuropathic chest pain [13]. The author described successful application in two severe neuropathic pain cases (metastatic disease to the ribs and malunion of rib fractures), with evidence of an extensive multi-dermatomal thoracic sensory block and supporting anatomical and radiological studies that highlighted the involvement of the dorsal and ventral branches of the thoracic spinal nerves. The author also included in this first fundamental report the application in postoperative or post-traumatic settings.

The application of ESP blocks has since expanded to encompass acute postoperative pain and various chronic pain conditions. Currently, there is a wealth of case reports documenting ESP block application at different spinal levels, along with RCTs and observational studies supporting its efficacy. In a case of 5 years of neuropathic pain, Fusco et al. highlighted the potential of erector spinae plane (ESP) block as a viable alternative to paravertebral block in chronic chest pain syndrome patients [14]. Subsequently, an observational study by Hochberg et al. [15] involving 110 patients with chronic pain of various etiologies examined the efficacy of ESP blocks ranging from T2 to T12. The results indicated that for 45 patients (41%), there was a reduction in NRS scores exceeding 50% of pre-procedural levels, with a mean follow-up duration of 7.9 ± 4.6 weeks.

For neuropathic pain, in addition to case reports, Lin et al. [16] conducted an RCT involving 52 patients suffering from post-herpetic neuralgia, demonstrating a reduction in post-herpetic neuralgia incidence at 12 weeks post-ESP block, along with decreased occurrences of neuropathic pain, sleep disturbances, and depression. Similarly, in an observational study, Aydin et al. [17] reported 34 patients suffering from acute and chronic herpetic pain, an immediate relief post-procedure, with sustained analgesic effects observed up to the third month and an NRS-score median of 1. Several reports, including those by Fusco et al. [14], Baixauli et al. [18], Kumar et al. [19], Jain et al. [20], Tulgar et al. [21], Fusco et al. [22], and Peksoz et al. [23] further corroborate the efficacy of ESP block in managing neuropathic pain.

ESP blocks have also demonstrated utility in cancer-related pain management. Studies by Unal Artik et al. [24], Aydin et al. [25], Ahiskalioglu et al. [26], Altiparmak et al. [27], Sirohiya et al. [28], and Jadon et al. [29] showcase successful applications of ESP blocks in various cancer types, including end-stage lung cancer, mesothelioma, breast cancer bone metastasis, Pancoast tumor, and advanced facial cancer.

In the context of chronic post-surgical pain, investigations into ESP blocks have yielded promising results. Zhang et al. [30] conducted an RCT involving 94 patients who underwent video-assisted lobectomy and reported significantly lower rates of postoperative chronic pain at 3 and 6 months in the ESP block group than in the general anesthesia alone group. Diaz-Bohada et al. [31], in an observational study including 42 patients who underwent VATS, reported that 35 patients who completed follow-up had a low incidence of chronic post-surgical pain and improved quality of life associated with ESP block.

ESP blocks have also been explored in breast surgery. Xin et al. [32] reported in 194 patients who a preoperative single-shot ESP combined with general anesthesia was not associated with a reduced incidence of chronic post-surgery pain (CPSP) at 1 year, although Piraccini et al. [33] and Hasoon et al. [34] previously reported effective treatment of post-mastectomy pain syndrome through ESP block and rhomboid intercostal block.

After right mini-thoracotomy, Toscano et al. [35] reported that the pain index was lower than 10 in 81/100 patients, and after coronary artery bypass grafting (CABG), Wiech et al. [36] reported that preemptive ESP block might decrease the risk of CPSP after the procedure.

Hasoon et al. [37] reported a case in which chronic pain following thoracotomy and video-assisted thoracic surgery (VATS) was successfully treated with ESP block. Several cases have been reported regarding the use of ESP block in post-thoracotomy pain syndrome (PTPS), a condition defined as pain recurring along the thoracotomy scar or persisting for more than 2 months after surgery. It has been estimated that PTPS affects 30–50% of patients (Forero et al. [38]; Piraccini et al. [39]; Sirohiya et al. [28]). An interesting report by Benkli et al. [40] discussed refractory post-surgical neuralgia after aneurysm rupture and craniotomy, as well as complex regional pain syndrome (CRPS) type 1 (Forero et al. [41]), with ESP catheter implantation under combined ultrasound and fluoroscopic guidance. A 2-year follow-up of continuous ESP showed an 80% reduction in pain scores from baseline and a 50% reduction in opioid consumption. Bang et al. [42] reported two other CRPS patients in which ESP catheterization proved to be effective for 14 days with breakthrough pain and decreased opioid consumption by 50%.

Of particular interest are ESP block implementation in headache: post-dural puncture headache (PDPH) (De Haan et al. [43]) and after craniotomy (Hernandez et al. [44]). Hong et al. [45] compared high thoracic ESP with cervical epidural injection for chronic shoulder pain in an RCT. A significant reduction in the NRS score was found in both groups, and the effect over time was statistically significant. Forero et al. [46] also reported the resolution of long-standing shoulder pain with a T3 ESP block, and Tulgar et al. [47] presented five cases of frozen shoulder pain treated with an ESP block.

For chronic myofascial pain, Tulgar et al. [21] first reported the application of ESP block to relieve lower cervical and interscapular myofascial pain as a new indication. Fusco et al. [48] hypothesized that (lumbar) ESP block combined with dry needling could provide effective analgesia for chronic thoracic pain, resulting in persistent pain reduction at the 4-month follow-up in a patient with burning interscapular pain. Yuruk et al. [49] demonstrated in an RCT with 60 patients who trapezius muscle injection (TMI) was superior to a single injection, but ESP block combined with TMI provided more effective analgesia than repeated TMI alone. ESP block with warm saline solution was used for treating chronic myofascial pain (Fusco et al. [50]), increasing sliding between fasciae, muscle relaxation, and pain improvement, as demonstrated through pre- and post-procedural elastography, which revealed decreased muscle stiffness and increased elasticity.

Two observational studies (Durmus et al. [51]; Merve Ata et al. [52], 96 + 43 patients in total) and one case series of ten consecutive patients (Gonçalves Morais et al. [53]) implemented lumbar ESP for chronic back pain, including radiculopathies. Lumbar ESP was also used for pain management in post-herpetic neuralgia (Kumar et al. [19]) and for post-herniorrhaphy pain at the L2 level, as well as for hip and low back pain after surgery (Takahashi et al. [54] and Canturk et al. [55]). Fusco et al. [56] described a case of chronic low back pain treated with lumbar ESP block, highlighting the dynamic technique of needle advancement together with local anesthetic injection to favor the opening of the fascial plane, which may contribute to disrupting the connective septa of the fascial compartment. Fascial changes are reported in chronic pain, potentially limiting physiologic fascial sliding and increasing muscle stiffness. Regarding the lumbar ESP injection technique, the authors of the cited works chose to release the mixture superficial to the transverse processes of the lumbar vertebrae along the undersurface of the erector spinal muscle; instead, some physicians cross the intertransverse ligaments with the needle and release the drug beneath the transverse processes [57]. A more recent application of the ESP block is the sacral approach. We found two case reports of sacral ESP application in chronic pain conditions: L5 radicular pain (Piraccini et al. [58]) and pelvic pain (vaginismus, Yilmaz et al. [59]).

### QL block

#### Literature review

Unlike the fascial plane blocks, this procedure represents an “intra-muscular” injection. Using the hyperechoic cortical bone of the iliac crest as a bony landmark, the needle is advanced inside the muscular belly of the QL with a technique that is different from the common fascial plane blocks [60]. At the time of this writing, there were 13 publications related to quadratus lumborum (QL) blocks, most of which reported positive outcomes. These included 1 systematic review and 4 randomized controlled trials (RCTs).

A systematic review by Du et al. [61] suggested the efficacy of QL blocks for postoperative analgesia following cesarean section, particularly in reducing acute postoperative pain, as evidenced by significantly decreased visual analog scale (VAS) scores and morphine consumption within the first 24 h after surgery. However, further research is necessary to assess its impact on managing chronic postoperative pain.

QL blocks have been utilized for various indications, including chronic hip pain in four patients (Fernandez Martin et al. [62]), chronic postoperative pelvic pain in a case report (Chutkowski et al. [63]), and abdominal neuropathic post-surgical pain (Carvalho et al. [64]). Observational studies have also been conducted. In a study involving 40 patients with chronic hip pain, a posterior QL block improved dynamic pain, particularly in patients with severe pain without significant joint injury and those with end-stage osteoarthritis (Fernandez Martin et al. [65]). Additionally, Niray et al. [66] showed that in 45 patients suffering from chronic flank pain, QL3 block may reduce opioid consumption at 6 months and emergency readmissions at 12 months.

Chronic post-surgical pain is a common issue following cesarean section. Borys et al. [67], in an observational prospective study of 233 patients, did not find that fascial blocks (such as the transversus abdominis plane or QL) improved post-surgical pain compared to standard treatment. However, in a subsequent RCT by Borys et al. [68] involving 105 patients, those who received QLB experienced significantly less persistent postoperative pain at months one and six following hospital discharge than those in the control group. Both the QLB and transversus abdominis plane (TAP) blocks were effective at managing pain after cesarean delivery, with the QLB potentially reducing the severity of persistent postoperative pain months after the procedure.

Regarding the same topic, the evidence is inconclusive: Mieszkowski et al. [69] conducted an RCT with 60 patients, all of whom received spinal anesthesia and were randomized to either the QLB group (receiving bilateral QL block type I) or the control group (receiving no block). They found no significant differences between the QLB and control groups regarding the occurrence of chronic post-surgical pain 6 months after cesarean section.

In another RCT by Chilkoti et al. [70] involving 60 patients, standard wound infiltration was compared to that of bilateral transversalis fascia plane block (TFPB). They found that the neuropathic pain component was significantly reduced and quality of life was improved in the block group.

Furthermore, in an RCT by Liu et al. [71], ultrasound-guided QLB provided superior short-term analgesia and reduced oxycodone consumption and the incidence of postoperative nausea and vomiting (PONV) after gastrointestinal surgery. However, this anesthetic technique did not significantly affect the incidence of chronic pain.

Another indication is the potential of QL blocks for treating myofascial chronic pain. We found two observational studies.

One study by Barreto Silva et al. [72] demonstrated that in 60 patients suffering from low back pain due to QL myofascial syndrome, infiltration of local anesthetic proved to be safe and effective within 6 months post-intervention.

The second study by Kongsagul et al. [73] included 142 patients with myofascial pain. The upper trapezius muscle (19.5%) was the most common muscle receiving the procedure, followed by the multifidus (10.0%) and quadratus lumborum (9.5%). The ultrasound-guided PSI technique showed a reduction in pain in 72.8% of the analyzed patients, with an acceptable pain relief period of 63 days.

## Anterior truncal blocks

### Pectoral nerve blocks

#### Literature review

Various studies have been performed to determine the efficacy of pectoral nerve blocks in chronic pain conditions. At the time, 23 publications were included, comprising two reviews and 5 RCTs.

One review by Avila et al. [74] analyzed neuropathic pain following breast cancer and reported that a serratus plane block, both superficial and deep, appears to be a viable option for post-mastectomy pain syndrome management, particularly in patients with specific symptoms such as tightness and severe neuropathic pain. The second review, by Ferreira-Silva et al. [75], consists of a technical review aiming to present an up-to-date summary of the most common ultrasound-guided fascial plane blocks for chronic pain in breast surgery patients.

The most important indication is persistent pain post-breast surgery (including mastectomy). The most commonly performed block in case reports was the serratus plane block (4 case reports, Piracha et al. [76], Takimoto et al. [77], Maranto et al. [78], and Liu et al. [79]). The results in terms of chronic post-mastectomy pain were similar except for one patient in the series by Liu, who reported no relief of tightness after the first injection but did report a small reduction in other neuropathic pain symptoms of “burning” and “stabbing.” Following the second injection 2 weeks later, she felt no further relief of any symptoms and elected not to return for the third control.

Five RCTs analyzed chronic pain after breast surgery; of these, one trial (Sulak et al. [80]) including 60 patients (with a serratus plane block versus saline) did not find an influence on quality of life. Moreover, no prevention of chronic post-mastectomy pain was demonstrated. Genc et al. [81] analyzed 90 patients in three study groups—ESP, pecto-serratus plane block, and control—and demonstrated that blocks had a modest effect on chronic pain scores. In 198 patients who underwent randomization to serratus anterior versus normal saline, Qjan et al. [82] reported that the incidence of CPSP decreased at 6 months from 37/89 to 17/90, with a relative risk of 0.72. Fuji et al. [83], in 80 patients allocated to the pectoral nerve 2 block or serratus plane, showed that compared with serratus plane block, the PECS-2 block reduced chronic pain 6 months after mastectomy. Flores et al. [84] conducted a specific follow-up in 49 patients based on a previous RCT. Twelve months after the surgical procedure, the blinded patients included in the previous study were interviewed by a blinded experimenter who found that a combination of general anesthesia with a serratus anterior block + PECS 1 reduced the occurrence of specific neuropathic pain descriptors and depressive symptoms.

Seven observational studies were included. A study by Perez-Herrero et al. [85] on chronic postoperative pain after breast surgery revealed that superficial anterior plane blocks of the serratus are effective and safe for pain control during the immediate postoperative period after breast cancer surgery as a part of the multimodal approach. No significant differences were found 1 week or 1 month after surgery.

Yang et al. [86], in 169 patients, reported that among responders, the mean pain relief duration was 45 days, with a median of 84 days, after serratus plane blocks (deep and superficial). Zocca et al. [87] analyzed only 8 patients who underwent superficial serratus plane block and showed that the initial improvement in symptoms ranged from 25% relief to near complete pain relief, with the duration of pain relief ranging from 2–3 days to 12 weeks. Fuzier et al. [88] described no persistent pain at 3 months in 69% of cases (of 137 patients). In a study by dos Santos et al. [89] involving 30 patients (deep serratus + parasternal blocks), a serratus block was shown to be a valid alternative for chronic pain during the first month when medical therapy was insufficient. De Cassai et al. [90], in 140 patients who underwent PECS + general anesthesia or general anesthesia alone, reported that the PECS group had a lower incidence of chronic pain at 3 months. Interestingly, a case study on the use of a serratus plane block for post-traumatic chronic pain (intercostal neuralgia) (Sir et al. [91]) and on the use of pectoral-intercostal fascial plane blocks and II-III and IV-V intercostal spaces for chronic post-sternotomy pain (Sahoo et al. [92]) was reported. Three observational studies on persistent pain after thoracic procedures were included: persistent post-surgical pain after VATS (Zhao et al. [93]) which showed that when SAPB was combined with continuous infusion of nonsteroidal anti-inflammatory drugs, no patient experienced moderate chronic pain for up to 3 months), post-thoracoscopy pain (Semyonov et al. [94] with the percentage of pain tending to be lower in the SAP group but not statistically significant) and chronic post-thoracic surgery pain (240 thoracotomies, 249 VATS total 489 patients, Okmen et al. [95]). In this study, the authors analyzed patients who underwent RIB and SAP block compared to those who underwent thoracic epidural and patient-controlled analgesia and found that the scores were significantly lower in the RIB and SAP block groups up to 6 months.

### TAP block

#### Literature review

At the time of this writing, there were 16 publications related to transverse abdominis plane block. No reviews are available. Only 3 RCTs were found. A trial by Theodoraki et al. [96] evaluating the short- and long-term postoperative analgesic efficacy of TAP block in inguinal hernia repair in 60 patients (TAP versus placebo) revealed that 6 months after surgery, pain scores at rest and during movement were low and comparable between the two groups, as the incidence of chronic pain was low and not significantly affected by the performance of the block. Post-inguinal hernioplasty chronic pain was induced in 72 patients who were randomized to receive subarachnoid (SAB group), general anesthesia (GA group), or subarachnoid block combined with a continuous TAP (TAP group). Pain and functional outcomes were assessed before and 6 months after surgery. Six months after surgery, the COMI-hernia score was lower in the TAP group than in the GA group or the SAB group. Adults with chronic abdominal wall pain who received trigger point injections reported significantly lower pain scores at the 3-month follow-up than patients who received a TAP block (Moeschler et al. [97]).

Chronic post-surgical pain is the principal indication for TAP block according to numerous case reports (Guirguis et al. [98] after laparoscopy; Gupta et al. [99], from multiple surgeries; Baciarello et al. [100], Sahoo et al. [101], Choudhary et al. [102], neuropathic post-herniorraphy) and observational studies (Paasch et al. [103] 1 year after hernia repair 289 patients included, Abd-Elsayed et al. [104] 92 patients and 30 patients [105]; Pan et al. [106], 307 patients and Sellam et al. [107], 44 patients). TAP block in all these studies was shown to provide some benefits, reducing the use of gabapentin or significantly improving abdominal pain scores for many weeks.

Anterior cutaneous nerve entrapment syndrome (ACNES), a condition that occurs when nerves within the abdominal wall—the anterior cutaneous abdominal nerves—become pinched or entrapped within the abdominal wall muscle, was reported as an indication for TAP block in reports by Nizamuddin et al. [108] and Sahoo [101].

An interesting application of TAP was in chronic pancreatitis (myofascial and visceral pain) (Niraj et al. [109]). In patients with myofascial pain secondary to chronic pancreatitis, the block was effective in producing clinically significant pain relief at 3 months (95%, 20/21) and durable pain relief lasting 6 months (62%, 13/21). In patients with visceral pain, the block produced a transient benefit lasting 2 to 3 weeks in one-third (6 of 17) of patients. A case of chronic abdominal pain in a healthy parturient patient was reported by Miller et al. [110].

## Lower extremities blocks

### FICB and PENG

#### Literature review

The literature on FIBC and PENG blocks is very limited. Kuo et al. [111] reported a fascia iliaca compartment block for osteosarcoma of the left proximal femur in a middle-aged female patient. One RCT by Diakomi et al. [112] evaluated the potential of preoperative fascia iliaca block in 182 patients to prevent chronic post-surgical pain after hip fracture repair under spinal anesthesia. This study proved that FICB in the perioperative setting may reduce the incidence, intensity, and severity of CPSP at 3 and 6 months after hip fracture surgery, providing safe and effective postoperative analgesia. Additionally, for the PENG block, one RCT by Guven Kose et al. [113] was found. This study aimed to compare the effectiveness of the pericapsular nerve group (PENG) block and intra-articular injection (IAI) of steroid-bupivacaine in the treatment of hip osteoarthritis (OA) in 60 patients suffering from chronic pain from osteoarthritis. PENG block provided effective pain relief, improving functionality and quality of life in hip OA patients for up to 2 months. Compared with those in the IAI group, the NRS scores in the PENG group (mean score = 2.57 ± 0.93, mean difference =  − 4.61) indicated significantly improved pain alleviation. Another observational study by Karaoglan et al. [114] in 112 patients with chronic hip pain evaluated pain at the first month and 3rd month of treatment following PENG block application. At the beginning of the 1st week, of the 112 patients who were administered a PENG block for hip pain, the authors reported a 62% improvement in pain, a 52% reduction in stiffness, and a 53% increase in physical activity. Even though these results slightly declined in the 1st and 3rd months, the rates were still higher than 45%. Server et al. [115] reported a case of successful PENG block placement for bilateral femoral avascular necrosis in an oncological patient. Multiple PENG blocks were applied, followed by five subsequent block applications at intervals of 2–4 weeks, and the pain finally stabilized at an NRS score of 1–2 in the case of osteoarthritic-related chronic hip pain (Sato et al. [116]).

### Adductor canal block

#### Literature review

The literature was limited to one RCT (chronic knee osteoarthritis pain) and 4 observational studies.

A trial by Ming et al. [117] evaluated the efficacy of adductor canal block (ACB) in comparison to intra-articular steroid-lidocaine injection (IASLI) in 66 patients with chronic knee osteoarthritis pain and demonstrated that ACB provides longer-lasting analgesia, which improves function and QoL in chronic KOA patients for up to 3 months without any significant side effects. A prospective study by Sreckovic et al. [118] in 166 patients with chronic post-surgical pain in the knee compared the effects of adductor canal block and IPACK on chronic post-surgical pain 2 years after TKA, showing that the combination of ACB and IPACK block provides adequate analgesia and reduces CPSP 2 years after surgery.

ACB appeared to decrease pain and increase ambulation in the study by Salihovic et al. [119].

In 35 patients with saphenous nerve entrapment and chronic knee pain, Taheri et al. [120] reported that the block is only moderately effective for controlling chronic knee pain. In 200 patients with medial knee pain from osteoarthritis, Lee et al. [121] showed at the 3-month follow-up that ACB is an effective and safe treatment and can be an option for patients who are either unresponsive or unable to take analgesics.

## Discussion

Since 2011, fascial blocks have represented an innovative and effective locoregional anesthesia modality facilitated by the widespread application of point-of-care ultrasound.

In this review, we sought studies evaluating the application of fascial blocks in chronic pain management, considering that, as a remedy for chronic pain, many fascial blocks were initially described and then applied in surgical and peri-operative contexts. For the selection of fascial blocks to include, we followed the approach of Kim et al. [122] and all considerations arising from recent top experts’ opinions [123]. In our review, we also included lower extremity fascial blocks such as PENG or FICB or adductor canal blocks whose application in chronic pain conditions is emerging.

According to the most comprehensive definition, the human fascial system can be defined as a three-dimensional continuum of soft and dense connective tissues that pervade the body, including various components such as adipose tissue, neurovascular sheaths, aponeuroses, the epineurium, joint capsules, ligaments, meninges, myofascial expansions, the periosteum, tendons, visceral fasciae and intra- and intermuscular connective tissues. This system encases all organs, muscles, bones, and nerves, providing the body with a structural framework and an environment conducive to integrated functioning [124].

In fascial blocks, unlike nerve or plexus blocks, local anesthetics are deposited in the anatomical compartment identified as the fascial plane, away from target nerves, via mechanisms linked to the administration of a large volume of local anesthetic solution to act on nerve fibers traveling inside the fascial plane or crossing through it. This may help avoid the potential for direct nerve trauma. Better analgesic outcomes arise when blocks are performed with higher volumes (15 to 30 ml in adults) because high volumes facilitate the spread of LA and the number of dermatomes involved. De Cassai et al. reported that the number of dermatomes reached by the anesthetic is closely related to the volume infused [125].

The use of high-volumes allows the fascial plane to open releasing the intra-fascial adhesions. This technique is well-described in the pertinent literature and is known as ultrasound-guided fascial hydro dissection. Indeed, the seeding technique is the best way to optimize the layer-by-layer dissection of the fascial planes restoring the intra-fascial gliding [126]. Associated with the use of large volume of injectate, there is always a potential risk of local anesthetic systemic toxicity (LAST). Several case reports of LASTs related to FPBs have been published, particularly when they are combined with other regional techniques or with catheters and intermittent or continuous infusions [127, 128]. Data about the drugs commonly used to perform the fascial plane blocks, e.g., saline solution, local anesthetic, steroids, or other adjuncts are reported in Table 6.

**Table 6:** Data about the drugs commonly used to perform the fascial plane blocks

**Different types of (ultrasound-guided) blocks**	**Drugs and dosages**
Posterior trunk	
***Thoracic erector spinae block (T-ESP)*** ***Lumbar erector spinae block (L-ESP)*** ***Sacral erector spinae block (S-ESP)*** ***Quadratus lumborum block (QL)***	*0.25%, 0.5% bupivacaine* *0.25% levobupivacaine* *0.1%, 0.1875%, 0.2%, 0.375%, 0.5% ropivacaine* *0.5%, 1% lidocaine, 1% prilocaine* *saline solution* *Adjuncts: methylprednisolone (40 or 80 mg), dexamethasone 2, 4, 3, 8, 10 mg, triamcinolone (10 or 40 mg) * *Volumes: 10–15–20–30 ml* *0.2% ropivacaine* *1% lidocaine* *0.25% levobupivacaine* *Adjuncts: dexamethasone 8 mg. methylprednisolone 20 mg* *Volumes: 15–25 ml*
Anterior trunk	
***P*** *ectoral nerve blocks* ***(PECS)*** ***Transversus abdominis plane block (TAP)***	*0.2%, 0.33%, 0.375% ropivacaine* *1% lidocaine* *0.25% levobupivacaine* *0.25%, 0.5% bupivacaine* *Adjuncts: dexamethasone 5 or 8 mg, methylprednisolone 20 or 40 mg, clonidine 50 mcg* *Volumes: 10–30 ml* *0.375%, 0.5%, 1% ropivacaine* *1% lidocaine* *0.25% bupivacaine* *Adjuncts: triamcinolone (40 mg), methylprednisolone (40 or 80 mg), epinephrine,* *Volumes: 20–30 ml*
Lower extremities	
***Fascia iliaca compartment block (FICB)*** ***Pericapsular nerve group block (PENG)*** ***Adductor canal block (ACB)***	*0.5% ropivacaine* *Adjuncts: dexamethasone (4 mg)* *Volumes: 20–40 ml* *0.25% bupivacaine,* *0.5% lidocaine* *0.5% ropivacaine* *Adjuncts: dexamethasone (4 or 8 mg)* *Volumes: 15–20 ml* *0.25% or 0.5% bupivacaine* *1% lidocaine* *0.25%, 0.33% levobupivacaine* *Adjuncts: methylprednisolone (40 mg),* *triamcinolone (10 mg), clonidine (100 mcg)* *Volumes: 10–20 ml*

Drugs and dosages used in the different blocks as emerging from included studies.

Chronic pain is not simply defined. It can be defined as pain persisting for more than 3 months [3], ranging from mild to severe intensity and originating from different injuries or illnesses of body tissues and/or of the somatosensory nervous system. The various types of chronic pain for which fascial blocks may be indicated (myofascial, neuropathic, post-surgical, oncologic) may not always have a clear classification. Diagnoses can be challenging, especially considering that the pathophysiological mechanisms of different types of pain can overlap. For this reason, our selection of studies and their classification according to pain type might be difficult.

Another field needing further investigation is the potential of regional anesthesia to prevent post-surgical chronic pain; however, in most surgical contexts, the benefit of locoregional anesthesia on persistent post-surgical pain has not yet been demonstrated [129]. For this reason, studies exploring the impact of preoperative fascial blocks in preventing the onset of post-surgical chronic pain syndromes were included in this review.

**Fig. 4:**
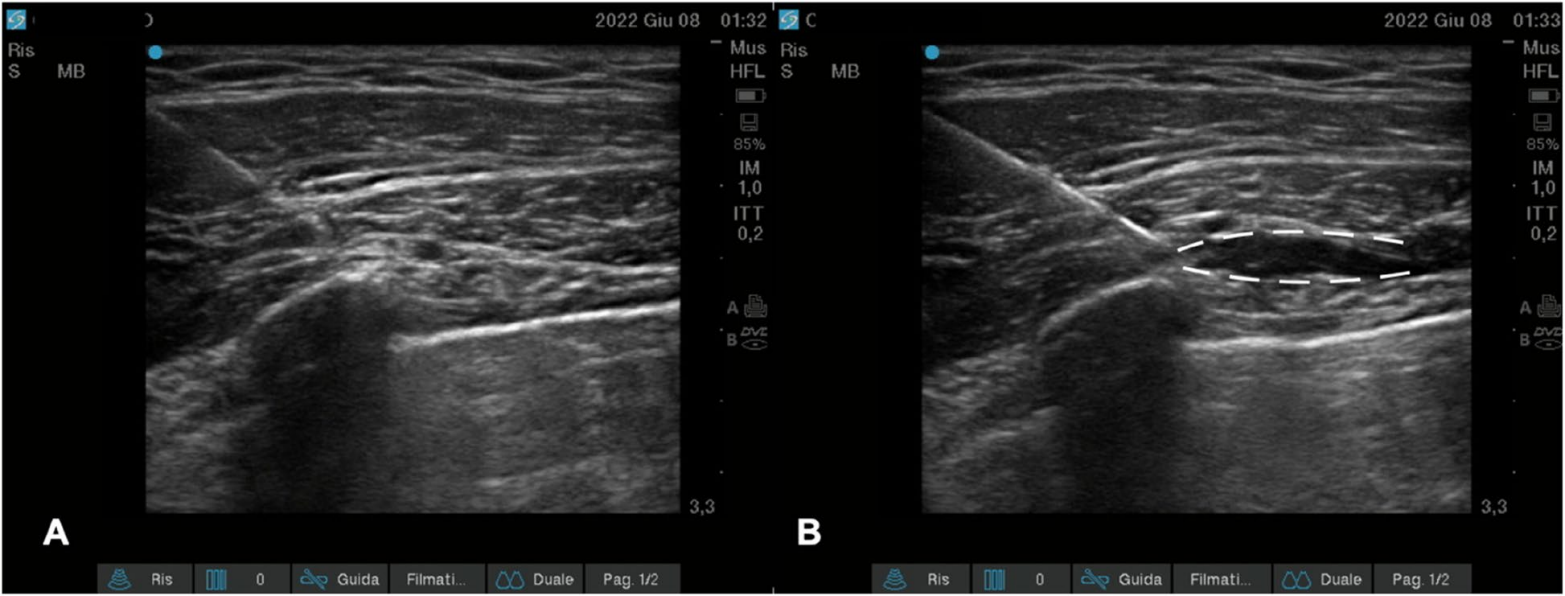
**A** Pecto-serratus plane block. **B** The Fusco V sign a sonographic marker for a successful interfascial plane block. The needle opens the fascial plane and local anesthetic is injected while the needle advances (dynamic V sign as another step to get a successful plane block)

With regard to our review, the greatest evidence arises from case report series, especially for certain blocks (such as erector spinae plane blocks), and only in more recent years have observational studies and randomized trials increased. Nevertheless, the best-documented studies revealed a significant tendency to support the use of fascial blocks in chronic painful conditions, especially in complex patients who are heavily influenced by the inefficacy of other treatments. There is, however, variability in the outcome and success of fascial blocks. The variability of fascial plane block outcomes may depend on various factors, which can be categorized into three groups: operator-related, patient-related, and drug-related. These factors are not always highlighted in all studies, especially in case reports. In this regard, the correct technique of injection-opening of the fascial plane can also contribute to determining the analgesic effect. An injection needle that opens the plane (the Fusco’s V sign) together with the dynamic insertion and injection of the solution helps to localize and open it, breaking down intra-fascial adhesions (Fig. 4).

It can therefore be risky to draw conclusions on indications considering the variability of the anatomical regions, the techniques and operators, the patients involved, and the drugs used. In many cases, patients need more than one treatment before achieving success. In other cases, the fascial plane blocks failed, or some patients refused long-term follow-up. Apart from failure, other side effects or complications were not reported.

Interestingly, in most cases, local anesthetics are enhanced by the use of an adjuvant, typically a corticosteroid, with no evidence of side effects or complications.

## Conclusions

Fascial plane blocks were initially based on a single ingenious, albeit flawed (as we will demonstrate), concept: the ability to block multiple (spinal) nerves traversing the spaces between muscles by administering high volumes of local anesthetics. The latest anatomical findings remind us that fascia itself should be considered a “potential pain generator”, as it is innervated and contains nerve fibers and corpuscles.

Interestingly, cadaveric and histological investigations have recently demonstrated the presence of multiple cutaneous branches of spinal nerves running inside the deep fascia of the cervical/thoracic spine. In this sense, the ultrasound-guided hydro dissection of this fascia can be considered a procedure to manage the fascial entrapment of these tiny nerves [130].

Moreover, its structure can undergo structural changes, impacting the mechanical function of the muscles it contains, as demonstrated by Fusco et al. through elastography. Changes in the stiffness and elasticity of tissues supported by fascia ultimately have significant repercussions on muscle trigger points and subsequent pain. An injection intervention into the fascia (including the cases of dry needling or saline solution or warm saline solution as well as appropriate solutions of a local anesthetic) thus also induces mechanical effects on the tissues, in addition to intercepting nerve endings. The ultrasound revolution facilitated the transition of this concept from theory to practical application. Most of the literature is filled with case series, case reports, technical briefs, and cadaver dye studies. The few randomized controlled trials that have been performed rarely compare plane blocks to a previous standard of care. Nevertheless, the fascial plane blocks in chronic pain management may represent a nuanced interplay between pharmacological efficacy, procedural precision, and affected person-focused care. As a quintessential issue of multimodal pain control strategies, fascial plane blocks together with other locoregional techniques could provide a centered, personalized approach to assuaging the weight of chronic pain, empowering patients to reclaim manipulation over their lives amidst adversity.

## Data Availability

No datasets were generated or analyzed during the current study.

## Abbreviations

ICD-11: International Classification of Diseases 11th Revision
FPBs: Fascial plane blocks
RCT: Randomized controlled trial
ESP: Erector spinae plane block
PECS 1: Interpectoral plane
PECS II: Pectoserratus plane
SAPB: Serratus anterior plane block
TAP: Transversus abdominis plane block
QL: Quadratus lumborum block
PENG: Pericapsular nerve group block
FICB: Fascia Iliaca compartment block
ACB: Adductor canal block
VAS: Visual analog scale
NRS: Numeric (Pain) Rating score
PRISMA: Preferred Reporting Items for Systematic Reviews and Meta-analyses
VATS: Video-assisted thoracoscopic surgery
PPSP: Persistent post-surgical pain
CPSP: Chronic post-surgical pain
CABG: Coronary artery bypass graft surgery
PTPS: Post-thoracotomy pain syndrome
CRPS: Complex regional pain syndrome
PDPH: Postdural puncture headache
TMI: Trapezius muscle injection
TFPB: Transversalis fascia plane block
PONV: Post-operative nausea and vomiting
PSI: Physiological saline injection
RIB: Rhomboid intercostal block
SAB: Spinal anesthesia block
GA: General anesthesia
ACNES: Anterior cutaneous nerve entrapment syndrome
IAI: Intra-articular injection
OA: Ostheoarthritis
IASLI: Intra-articular steroid-lidocaine injection
KOA: Knee ostheo-arthritis
IPACK: Interspace Between the Popliteal Artery and Capsule of the Knee
TKA: Total knee arthroplasty
LAST: Local anesthetic systemic toxicity
ERAS: Enhanced recovery after surgery
LA: Local anesthetic
GRADE: Grades of Recommendation, Assessment, Development and Evaluation
